# ULBP1 is induced by hepatitis C virus infection and is the target of the NK cell‐mediated innate immune response in human hepatocytes

**DOI:** 10.1002/2211-5463.12373

**Published:** 2018-01-25

**Authors:** Hiromichi Dansako, Hirotaka Imai, Youki Ueda, Shinya Satoh, Takaji Wakita, Nobuyuki Kato

**Affiliations:** ^1^ Department of Tumor Virology Okayama University Graduate School of Medicine, Dentistry and Pharmaceutical Sciences Okayama Japan; ^2^ Department of Virology II National Institute of Infectious Disease Tokyo Japan

**Keywords:** HCV RNA replication, hepatitis C virus, innate immune response, NK cell, ULBP1

## Abstract

Natural killer (NK) cells through their NK group 2 member D (NKG2D) receptors recognize NKG2D ligands such as UL16‐binding proteins (ULBPs) on virus‐infected cells and subsequently trigger the host innate immune response. In the present study, we demonstrated that hepatitis C virus (HCV) induced the cell surface expression of ULBP1 in human immortalized hepatocyte PH5CH8 cells and human hepatoma HuH‐7 cell‐derived RSc cells. Interestingly, NK cell line NK‐92 induced cytotoxicity and interferon‐γ mRNA expression and subsequently reduced the levels of HCV RNA replication during co‐culture with HCV‐infected RSc cells. From these results, we conclude that ULBP1 is a target of the NK cell‐mediated innate immune response in HCV‐infected human hepatocytes.

**Abbreviations**
ADR: adriamycin
GAPDH: glyceraldehyde 3‐phosphate dehydrogenase
HCMV: human cytomegalovirus
HCV: hepatitis C virus
IFN: interferon
MICA: MHC class I chain‐related A
MICB: MHC class I chain‐related B
NKG2A: NK group 2 member A
NKG2D: NK group 2 member D
NK: natural killer
NS2: non‐structural protein 2
RAET1G: retinoic acid early transcript 1G
sMICA: soluble forms of MICA
sMICB: soluble forms of MICB
sULBP2: soluble forms of ULBP2
ULBP: UL16‐binding protein
UV‐JFH‐1: ultraviolet‐inactivated JFH‐1

Hepatitis C virus (HCV) is an enveloped single‐stranded RNA virus and a member of the *Flaviviridae* family 1. Persistent HCV infection causes chronic hepatitis, which often leads to liver cirrhosis and hepatocellular carcinoma 2. These hepatic diseases are tightly associated with an excessive inflammatory response to persistent HCV infection in the liver. Therefore, to prevent the progression of hepatic diseases, it is necessary to inhibit the inflammatory response to persistent HCV infection.

In viral infection, the host initiates inflammation through an innate immune response. Natural killer (NK) cells are known to play important roles in the host innate immune response to viral infection 3. NK cells express a variety of activating and inhibitory receptors on their surface 4. Under steady‐state conditions, the activation of NK cells is inhibited by a signal through the inhibitory receptors to prevent the NK cells from attacking normal cells. However, in the presence of viral infection, NK cells are activated by a signal through activating receptors such as NK group 2 member D (NKG2D). NKG2D interacts with its ligands (NKG2D ligands) on virus‐infected cells, and subsequently triggers the activating signal to attack virus‐infected cells. Thus, NK cells discriminate between the normal cells and virus‐infected cells through the interaction of NKG2D with NKG2D ligands on virus‐infected cells.

During viral infection, the expression of NKG2D is modulated on NK cells. With respect to HCV, the expression of NKG2D has been reported to increase in the acute phase of both patients with chronic infection and patients with self‐limited infection 5, 6, 7. In contrast, other groups have reported that the expression of NKG2D is not changed in patients with chronic HCV infection 8, 9. Due in part to these conflicting results, the roles of NKG2D in the host innate immune response to HCV infection remain uncertain.

The roles of NKG2D ligands during HCV infection also remain uncertain. In humans, NKG2D ligands include UL16‐binding proteins (ULBP) 1–4, retinoic acid early transcript 1G (RAET1G/ULBP5) and MHC class I chain‐related A and B (MICA and MICB) 10. These NKG2D ligands are induced by the stress‐associated pathway and oncogene‐driven pathway. The DNA damage response has been reported to induce the expression of ULBP1, ULBP2 and ULBP3 in human foreskin fibroblasts 11. On the other hand, viral infection has been shown to induce NKG2D ligands 12, 13. Human cytomegalovirus (HCMV) infection induced ULBP1, ULBP2 and ULBP3, but HCMV glycoprotein UL16 inhibited NKG2D‐mediated recognition by its binding with ULBP1 and ULBP2 in human foreskin fibroblasts 12. HIV‐1 infection induced the surface expression of ULBP1 and ULBP2 but not ULBP3, MICA or MICB in primary CD4^+^ T‐cells through the DNA damage response 13. In the present study, in order to understand how HCV triggers host innate immune response through NK cells, we attempted to identify the NKG2D ligands induced by HCV infection.

## Materials and methods

### Cell culture and reagents

Human immortalized hepatocyte PH5CH8 cells 14 and human hepatoma HuH‐7 cell‐derived RSc cells 15 were cultured as previously described 16. The NK cell line NK‐92 17 was purchased from the American Type Culture Collection (ATCC; Manassas, VA, USA) and cultured according to the manufacturer's instructions. NK‐92 cells were previously reported to induce interferon (IFN)‐γ through cell‐to‐cell contact with influenza A or Sendai virus‐infected macrophages 18. In addition, NK‐92 cells were reported to augment the cytotoxicity against Newcastle disease virus‐infected cells 19.

The DNA‐damaging agent adriamycin (ADR) was purchased from Sigma‐Aldrich (St Louis, MO, USA). The synthetic dsRNA analog poly IC was purchased from Invivogen (San Diego, CA, USA).

### Construction of expression vectors

The pCX4bleo/ULBP1 retroviral vector was constructed by the introduction of *ULBP1* (accession no. NM_025218) cDNA containing a full‐length ORF into the pCX4bleo retroviral vector 20 as previously described 21. The pCX4pur/C‐NS2 (O) and pCX4bsr/C‐NS2 (JFH‐1) retroviral vectors were constructed by the introduction of the region encoded from core to non‐structural protein 2 (NS2) (O strain or JFH‐1 strain) into the pCX4pur 20 or pCX4bsr 20 retroviral vector, respectively. The pCX4bsr/ NS3‐5B (O) and pCX4pur/NS3‐5B (JFH‐1) retroviral vectors were also constructed by the introduction of the region encoded from NS3 to NS5B (O strain or JFH‐1 strain) into the pCX4bsr or pCX4pur retroviral vector, respectively. These expression vectors were used for the generation of PH5CH8 ULBP1 cells, PH5CH8 C‐NS2&NS3‐5B (O) cells and PH5CH8 C‐NS2&NS3‐5B (JFH‐1) cells, respectively.

### Co‐culture of the target cells with NK‐92 cells

Before the co‐culture with NK‐92 cells, the culture medium of target cells was replaced with fresh medium. Subsequently, an equal volume of fresh medium containing NK‐92 cells was added to the target cells. The target cells were co‐cultured with NK‐92 cells for 2, 6 or 24 h in a 1 : 1 mixed medium (two kinds of media for each cell type). At 2, 6 or 24 h after co‐culture using several different E : T ratios, the culture media were recovered from the co‐cultured cells for the measurement of NK cell‐mediated cytotoxicity as described below.

For the blocking of NKG2D ligands, target cells were also treated with anti‐ULBP1 rabbit polyclonal antibody (GTX123021; GeneTex, Irvine, CA, USA) or anti‐ULBP2/5/6 antibody (R&D Systems, Minneapolis, MN, USA) for an hour before the co‐culture with NK‐92 cells. Subsequently, NK cell‐mediated cytotoxicity was measured as described below.

### Quantitative RT‐PCR analysis

Isolation of total RNA from cells and quantitative RT‐PCR analysis were performed as previously described 22. For quantitative RT‐PCR analysis, we used primer sets previously described for glyceraldehyde 3‐phosphate dehydrogenase (GAPDH) 22, HCV 22, ULBP3 23, ULBP4 23, RAET1G/ULBP5 23, MICA 23 and MICB 23. We also prepared the following forward and reverse primer sets: for ULBP1, 5′‐GGATCCAACAAAACCACCCTCTC‐3′ (forward) and 5′‐GGACCCAGACCAGGCTAACAG‐3′ (reverse); for ULBP2, 5′‐GCTACCAAGATCCTTCTGTGC‐3′ (forward) and 5′‐AGAGAGTGAGGGTCGGCTC‐3′ (reverse); for IFN‐γ, 5′‐TGGCTTTTCAGCTCTGCATCG‐3′ (forward) and 5′‐TTTCTGTCACTCTCCTCTTTCCA‐3′ (reverse); and for NK group 2 member A (NKG2A), 5′‐GTCTGCGAAGATTGCAGGCAT‐3′ (forward) and 5′‐TGTCAGGGACTGTACTCTTCTGTC‐3′ (reverse). The expression levels were normalized to those of GAPDH or NKG2A. The mean value and the standard deviation were calculated from three independent experiments.

### Western blot analysis

SDS/PAGE and subsequent detection of immunocomplex were performed as previously described 24. Anti‐Core (CP11; Institute of Immunology Co., Japan), anti‐phospho‐Chk2 (Thr68) (Cell Signaling Technology, Beverly, MA, USA), anti‐Chk2 (Cell Signaling Technology) and anti‐β‐actin (AC‐15; Sigma‐Aldrich) antibodies were used as primary antibodies.

### Flow cytometric analysis

Intracellular and cell surface ULBP1s were detected by a flow cytometer. Briefly, after digitonin‐permeabilization of cells, intracellular ULBP1 was immunostained by using anti‐ULBP1 rabbit polyclonal antibody (GTX123021; GeneTex) and PE‐conjugated goat anti‐rabbit antibody (Jackson ImmunoResearch Laboratories, West Grove, PA, USA) as primary and secondary antibody, respectively. Cells without digitonin‐permeabilization were also subjected to the immunostaining of cell surface ULBP1. Rabbit IgG isotype control (Cell Signaling Technology) was used as a negative control. The mean fluorescence intensity and the standard deviation were calculated from four independent experiments.

The binding of NKG2D to target cells was also detected by a flow cytometer. Briefly, target cells were subjected to the treatment of recombinant human NKG2D/Fc (R&D Systems) or human IgG_1_/Fc chimera protein (R&D Systems), and then were immunostained by FITC‐conjugated goat anti‐human IgG, Fc fragment specific (Jackson ImmunoResearch Laboratories). The mean fluorescence intensity and the standard deviation were calculated from three independent experiments.

### Measurement of NK cell activity

NK cell activity was assessed by the cell viability of the target cells and the cytotoxicity to the target cells. At 24 h after the co‐culture of the target cells with NK‐92 cells, the surviving cells were stained with Coomassie brilliant blue. NK cell‐mediated cytotoxicity was measured by a lactate dehydrogenase cytotoxicity detection kit (TaKaRa, Kusatsu, Japan) after the co‐culture. The level of cytotoxicity was calculated relative to that in the target cells with Triton X‐100 treatment, which was set at 100%.

### Statistical analysis

Student's *t* test was used for the determination of significant differences among groups. Values of *P *<* *0.05 were considered to indicate statistical significance.

## Results

### The expression of HCV proteins enhances the levels of intracellular ULBP1 in human immortalized hepatocyte PH5CH8 cells

**Figure 1:**
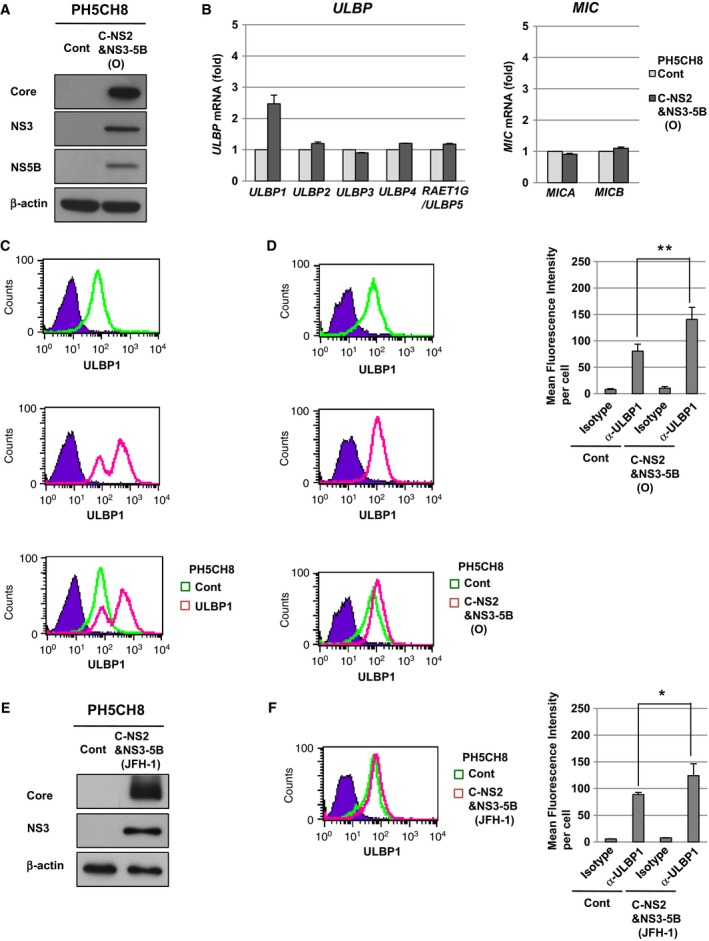
HCV proteins enhanced intracellular ULBP1 in PH5CH8 cells. (A) Western blot analysis of HCV proteins in PH5CH8 C‐NS2&NS3‐5B (O) cells. (B) Quantitative RT‐PCR analysis of the mRNA of NKG2D ligands in PH5CH8 C‐NS2&NS3‐5B (O) cells. The level of each NKG2D ligand in PH5CH8 C‐NS2&NS3‐5B (O) cells was calculated relative to that in PH5CH8 Cont cells, which was set at 1. (C) Flow cytometric analysis of intracellular ULBP1 in PH5CH8 ULBP1 cells. Signals of intracellular ULBP1 in PH5CH8 Cont and ULBP1 cells are shown in green and pink, respectively. An isotype control was used as a negative control (violet area). (D) Flow cytometric analysis of intracellular ULBP1 in PH5CH8 C‐NS2&NS3‐5B (O) cells. Signals of intracellular ULBP1 in PH5CH8 Cont and C‐NS2&NS3‐5B (O) cells are shown in green and pink, respectively (left panels). The mean fluorescence intensity and the standard deviation were calculated from four independent experiments (right graph). **P < 0.01. (E) Western blot analysis of HCV proteins in PH5CH8 C‐NS2&NS3‐5B (JFH‐1) cells. (F) Flow cytometric analysis of intracellular ULBP1 in PH5CH8 C‐NS2&NS3‐5B (JFH‐1) cells. Signals of intracellular ULBP1 in PH5CH8 Cont and C‐NS2&NS3‐5B (JFH‐1) cells are shown in green and pink, respectively (left panels). The mean fluorescence intensity and the standard deviation were calculated from four independent experiments (right graph). *P < 0.05.

NK cells discriminate between normal cells and target cells through the recognition of NKG2D ligands on the target cells. NKG2D ligands are the target molecules of NK cells, and are enhanced in response to cellular stresses such as DNA damage on the target cells 11. HCV core protein and NS3 protein are reported to induce DNA damage in hepatocytes through DNA double‐strand breaks 25. On the other hand, we previously reported that HCV core protein might disturb the DNA repair system by promoting microsatellite instability in human immortalized hepatocyte PH5CH8 cells, which have a non‐neoplastic phenotype 26. In addition, we demonstrated that HCV NS5B protein increased the susceptibility to DNA double‐strand breaks in PH5CH8 cells 27. Thus, we conjectured that HCV would enhance NKG2D ligands through a DNA damage response. To investigate this possibility, we first examined whether HCV affected the expression of NKG2D ligands by using PH5CH8 cells. Since PH5CH8 cells previously showed low susceptibility to HCV infection 14, we prepared PH5CH8 cells stably expressing HCV proteins (O strain, genotype 1b) as the target cells (designated PH5CH8 C‐NS2&NS3‐5B (O) cells; Fig. 1A). Among NKG2D ligands, only *ULBP1* mRNA was significantly increased in PH5CH8 C‐NS2&NS3‐5B (O) cells (Fig. 1B). We next examined the intracellular expression of endogenous ULBP1 in PH5CH8 C‐NS2&NS3‐5B (O) cells by flow cytometric analysis. The results showed that, in addition to the stable, exogenous expression of ULBP1 in PH5CH8 cells (designated PH5CH8 ULBP1 cells, Fig. 1C), the intracellular level of ULBP1 was also significantly enhanced in PH5CH8 C‐NS2&NS3‐5B (O) cells compared with PH5CH8 Cont cells (Fig. 1D). Finally, the intracellular level of ULBP1 was significantly enhanced in PH5CH8 C‐NS2&NS3‐5B (JFH‐1) cells (JFH‐1 strain, genotype 2a; Fig. 1E,F). These results suggested that HCV proteins enhanced the levels of intracellular ULBP1 in PH5CH8 cells.

### The expression of HCV proteins enhances the cell surface expression of ULBP1 in PH5CH8 cells

**Figure 2:**
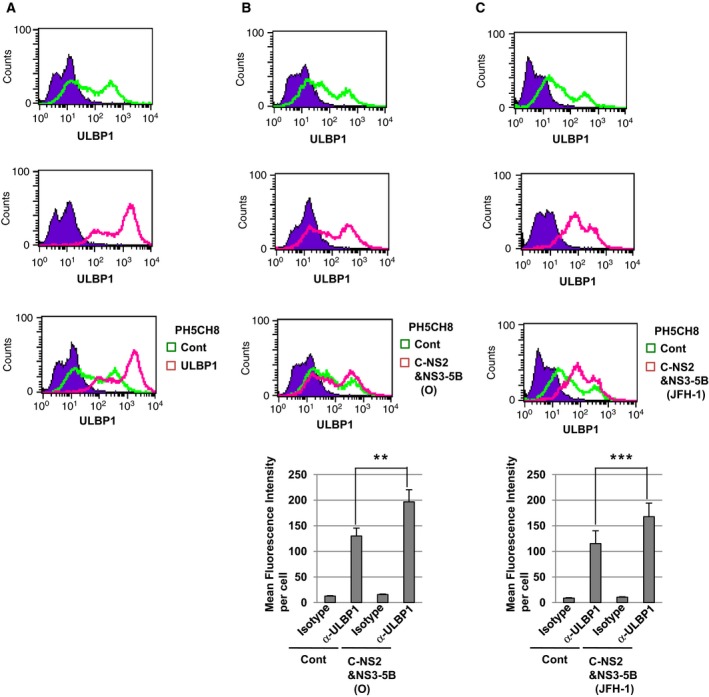
HCV proteins enhanced the cell surface expression of ULBP1 in PH5CH8 cells. Flow cytometric analysis of the cell surface ULBP1 in (A) PH5CH8 ULBP1 cells, (B) PH5CH8 C‐NS2&NS3‐5B (O) cells and (C) PH5CH8 C‐NS2&NS3‐5B (JFH‐1) cells. Signals of the cell surface ULBP1 in PH5CH8 Cont are shown in green. Signals of the cell surface ULBP1 in PH5CH8 ULBP1, C‐NS2&NS3‐5B (O), or C‐NS2&NS3‐5B (JFH‐1) cells are shown in pink. An isotype control was used as a negative control (violet area). Bar graphs: the mean fluorescence intensity and the standard deviation were calculated from four independent experiments. **P < 0.01, ***P < 0.001.

NK cells are known to recognize NKG2D ligands presented at the surface of target cells. Our results showed that HCV proteins enhanced intracellular ULBP1 in PH5CH8 cells (Fig. 1D,F). We next examined the cell surface expression of ULBP1 in PH5CH8 C‐NS2&NS3‐5B (O) cells and PH5CH8 C‐NS2&NS3‐5B (JFH‐1) cells by flow cytometric analysis. In PH5CH8 ULBP1 cells, the expression of exogenous ULBP1 was enhanced at the cell surface (Fig. 2A) as well as in the intracellular compartment (Fig. 1C). On the other hand, the expression of endogenous ULBP1 was significantly enhanced at the surface of PH5CH8 C‐NS2&NS3‐5B (O) cells (Fig. 2B) and PH5CH8 C‐NS2&NS3‐5B (JFH‐1) cells (Fig. 2C). These results suggested that HCV proteins enhanced the cell surface expression of ULBP1 in PH5CH8 cells.

### The human NK cell line NK‐92 exhibits cytotoxicity through the recognition of ULBP1 at the surface of PH5CH8 cells

**Figure 3:**
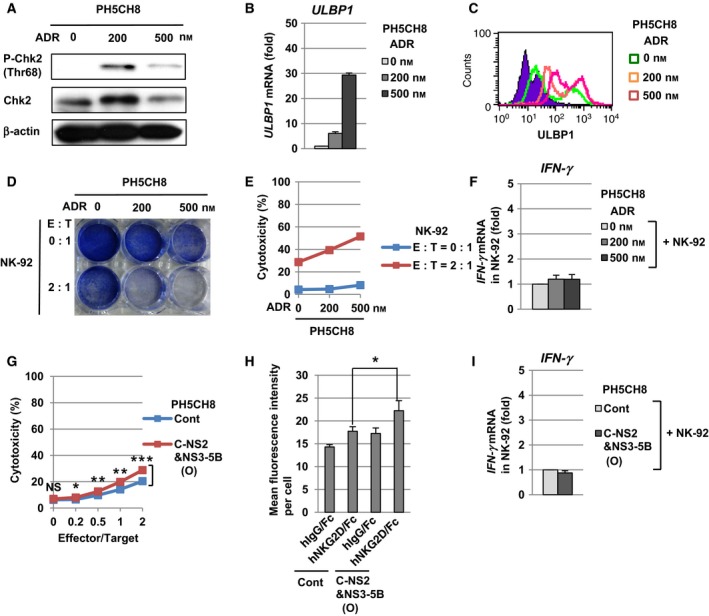
NK‐92 cells exhibited cytotoxicity through the recognition of ULBP1 at the surface of HCV protein‐expressing PH5CH8 cells. (A) Western blot analysis of phospho‐Chk2 (Thr68) in ADR‐treated PH5CH8 cells. (B) Quantitative RT‐PCR analysis of *ULBP1* mRNA in ADR‐treated PH5CH8 cells. The level of *ULBP1* mRNA in ADR‐treated PH5CH8 cells was calculated relative to that in PH5CH8 cells without ADR treatment, which was set at 1. (C) Flow cytometric analysis of the cell surface ULBP1 in ADR‐treated PH5CH8 cells. An isotype control was used as a negative control (violet area). (D) Cell viability assay in ADR‐treated PH5CH8 cells after the co‐culture with NK‐92 cells. The surviving cells were stained with Coomassie brilliant blue. (E) Measurement of NK‐92‐mediated cytotoxicity towards ADR‐treated PH5CH8 cells by a lactate dehydrogenase cytotoxicity detection kit. (F) Quantitative RT‐PCR analysis of *IFN‐*γ mRNA in NK‐92 cells after the co‐culture with ADR‐treated PH5CH8 cells. Total RNA was isolated from NK‐92 cells at 24 h after the co‐culture. (G) Measurement of NK‐92‐mediated cytotoxicity towards PH5CH8 C‐NS2&NS3‐5B (O) cells. NS, not significant. *P < 0.05, **P < 0.01, ***P < 0.001. (H) Flow cytometric analysis of the binding of NKG2D/Fc chimera proteins to PH5CH8 C‐NS2/NS3‐5B (O) cells. *P < 0.05. (I) Quantitative RT‐PCR analysis of *IFN‐*γ mRNA in NK‐92 cells after the co‐culture with PH5CH8 C‐NS2&NS3‐5B (O) cells.

Our results showed that HCV proteins enhanced the cell surface expression of ULBP1 in PH5CH8 cells (Fig. 2B,C). To examine NK cell‐mediated recognition through ULBP1, we next measured the cell viability, the cytotoxicity and IFN‐γ induction during the co‐culture of target cells with the NK cell line NK‐92. As the target cells of NK‐92 cells, we first prepared PH5CH8 cells treated with a DNA‐damaging agent, ADR. In PH5CH8 cells, ADR treatment induced phosphorylation of Chk2 at threonine 68, suggesting that caused the DNA damage response (Fig. 3A). The ADR‐triggered DNA‐damage response caused the enhancement of *ULBP1* mRNA (Fig. 3B), and subsequently the expression of ULBP1 at the cell surface (Fig. 3C) in a dose‐dependent manner. At 24 h after co‐culture at an effector‐to‐target (E : T) ratio of 2 : 1, NK‐92 cells decreased the cell viability of ADR‐treated PH5CH8 cells (Fig. 3D). Consistent with this result, NK‐92 cells enhanced their cytotoxicity against ADR‐treated PH5CH8 cells (Fig. 3E). On the other hand, we did not observe that NK‐92 cells induced *IFN‐*γ mRNA against ADR‐treated PH5CH8 cells (Fig. 3F). These results suggested that the NK cells targeted ADR‐treated PH5CH8 cells through the recognition of cell surface ULBP1, and then attacked PH5CH8 cells via their cytotoxicity. We next examined whether NK‐92 cells also recognized ULBP1 enhanced at the surface of PH5CH8 C‐NS2&NS3‐5B (O) cells. At 24 h after the co‐culture with PH5CH8 C‐NS2&NS3‐5B (O) cells at an E : T ratio of 2 : 1, NK‐92 cells significantly enhanced the cytotoxicity (Fig. 3G). Consistent with this result, the binding of NKG2D/Fc fusion protein to PH5CH8 C‐NS2&NS3‐5B (O) cells was significantly enhanced compared to that of PH5CH8 Cont cells (Fig. 3H). On the other hand, we did not observe that NK‐92 cells induced the production of *IFN‐*γ mRNA against PH5CH8 C‐NS2&NS3‐5B (O) cells (Fig. 3I). These results suggested that NK cells targeted HCV protein‐expressing PH5CH8 cells through the recognition of ULBP1 on their cell surfaces.

### HCV infection enhances both NK cell‐mediated cytotoxicity and IFN‐γ induction through the cell surface expression of ULBP1 in RSc cells

**Figure 4:**
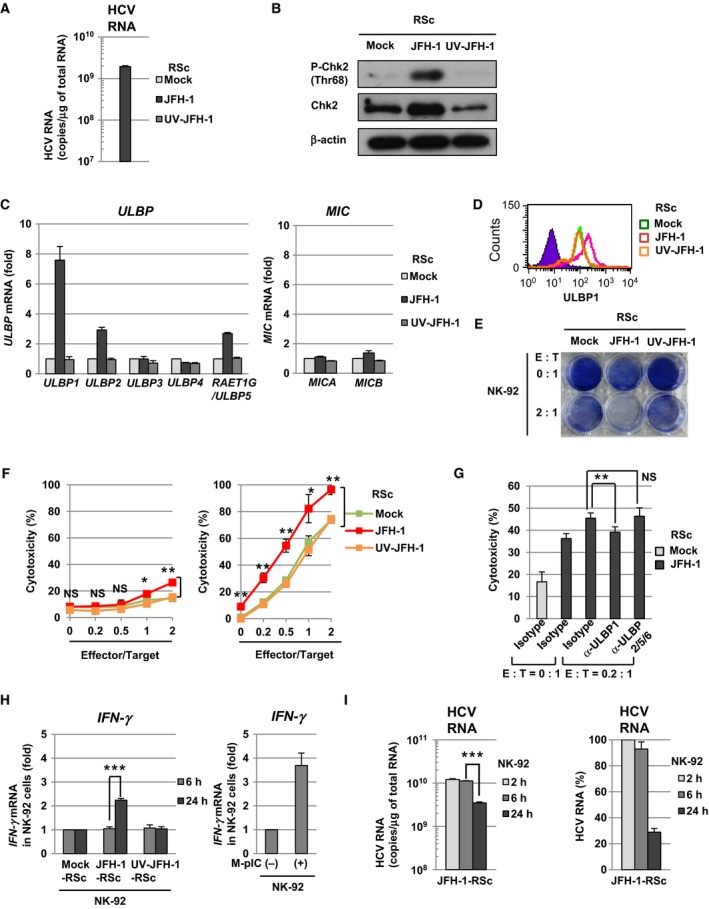
HCV infection enhances both NK cell‐mediated cytotoxicity and IFN‐γ induction through the cell surface expression of ULBP1 in RSc cells. (A) Quantitative RT‐PCR analysis of HCV RNA in RSc cells 72 h after infection with JFH‐1 at a multiplicity of infection of 1. (B) Western blot analysis of phospho‐Chk2 (Thr68) in JFH‐1‐RSc cells. (C) Quantitative RT‐PCR analysis of the mRNA of NKG2D ligands in JFH‐1‐RSc cells. Each level of NKG2D ligands in JFH‐1‐RSc cells was calculated relative to that in mock‐RSc cells, which was set at 1. (D) Flow cytometric analysis of the cell surface ULBP1 in JFH‐1‐RSc cells. An isotype control was used as a negative control (violet area). (E) Cell viability assay in JFH‐1‐RSc cells after co‐culture with NK‐92 cells. (F) Measurement of NK‐92‐mediated cytotoxicity towards JFH‐1‐RSc cells at 6 h (left) or 24 h (right) after the start of co‐culture. NS, not significant. *P < 0.05, **P < 0.01. (G) Functional analysis of ULBP1 on NK‐92‐mediated cytotoxicity towards JFH‐1‐RSc cells. NS, not significant. **P < 0.01. (H) Left: quantitative RT‐PCR analysis of *IFN‐*γ mRNA in NK‐92 cells after co‐culture with JFH‐1‐RSc cells. ***P < 0.001. Right: quantitative RT‐PCR analysis of *IFN‐*γ mRNA in NK‐92 cells treated poly IC. (I) Left: quantitative RT‐PCR analysis of HCV RNA in JFH‐1‐RSc cells after co‐culture with NK‐92 cells. ***P < 0.001. Right: from the results of the left panel, the HCV RNA level was calculated relative to that in JFH‐1‐RSc cells at 2 h after co‐culture with NK‐92 cells, which was set at 100%.

HCV infection is reported to induce DNA damage in hepatocytes through double‐strand breaks 25. Therefore, we next examined whether HCV infection would enhance the cell surface expression of ULBP1 through the DNA damage response in RSc cells derived from the human hepatoma cell line HuH‐7. Our previous results demonstrated that the susceptibility to HCV infection in RSc cells was almost comparable to that in Huh7.5 cells 15. As shown in ADR‐treated PH5CH8 cells (Fig. 3C), HCV infection also induced phosphorylation of Chk2 at threonine 68 in RSc cells, suggesting that caused DNA damage response (Fig. 4A,B). HCV infection‐mediated DNA damage response increased the levels of *ULBP1*,* ULBP2* and *ULBP5* mRNA in RSc cells (Fig. 4C). Among NKG2D ligands, only *ULBP1* mRNA was increased in both HCV protein‐expressing PH5CH8 cells (Fig. 1B) and HCV‐infected RSc cells (Fig. 4C). Moreover, as shown in HCV protein‐expressing PH5CH8 cells (Fig. 2B,C), the cell surface expression of ULBP1 was also enhanced in HCV‐infected RSc cells (Fig. 4D). From these results, we considered that HCV‐infected cells would be targeted through NK cell‐mediated recognition of ULBP1. To examine NK cell‐mediated recognition of ULBP1, we also measured the cell viability, the cytotoxicity and the IFN‐γ induction during a co‐culture of JFH‐1‐infected RSc cells with NK‐92 cells. At 24 h after the start of co‐culture at an E : T ratio of 2 : 1, the viability of JFH‐1‐infected RSc cells was lower than that of mock‐ or ultraviolet‐inactivated JFH‐1 (UV‐JFH‐1)‐infected RSc cells (Fig. 4E). Consistent with this result, the NK‐92 cell‐mediated cytotoxicity against JFH‐1‐infected RSc cells was higher than that against mock‐ or UV‐JFH‐1‐infected RSc cells at both 6 h and 24 h after the start of co‐culture at an E : T ratio of 2 : 1 (Fig. 4F). In addition, anti‐ULBP1 but not anti‐ULBP2/5/6 antibody significantly inhibited NK‐92 cell‐mediated cytotoxicity against JFH‐1‐infected RSc cells (Fig. 4G). These results suggested that HCV infection enhanced the susceptibility towards NK cell‐mediated cytotoxicity through the cell surface expression of ULBP1 in RSc cells. Interestingly, NK‐92 cells induced *IFN‐*γ mRNA against JFH‐1‐infected RSc cells but not mock‐ or UV‐JFH‐1‐infected RSc cells at 24 h after the start of co‐culture at an E : T ratio of 2 : 1 (Fig. 4H, left panel). The addition of the synthetic dsRNA analog (poly IC) to the culture media (M‐pIC) also induced *IFN‐*γ mRNA in NK‐92 cells (Fig. 4H, right panel). These results suggested that HCV dsRNA (viral replicative intermediate) was released from JFH‐1‐infected RSc cells by NK‐92 cell‐mediated cell lysis and subsequently triggered *IFN‐*γ mRNA induction in NK‐92 cells. Consistent with this result, the levels of HCV replication in surviving JFH‐1‐infected RSc cells decreased at 24 h after the start of co‐culture with NK‐92 cells at an E : T ratio of 2 : 1 (Fig. 4I). These results suggested that NK cells targeted HCV‐infected cells through the recognition of cell surface ULBP1 and subsequently inhibited these cells via both NK cell‐mediated cytotoxicity and IFN‐γ induction.

## Discussion

NK cells play important roles in the host innate immune response to viral infection. Upon viral infection, NK cells are activated by a signal initiated by the interaction of their NKG2D with NKG2D ligands on virus‐infected cells. Among several known NKG2D ligands, HCMV infection was shown to induce ULBP1, ULBP2 and ULBP3 in human foreskin fibroblasts 12. HIV‐1 infection also induced the surface expression of ULBP1 and ULBP2 in primary CD4^+^ T‐cells through the DNA damage response 13. In the present study, we demonstrated that HCV induced the surface expression of ULBP1 in both PH5CH8 cells (Fig. 2B,C) and RSc cells (Fig. 4D). However, we have not identified which HCV protein is responsible for the surface expression of ULBP1 through the DNA damage response. Another group previously suggested that HCV core protein or NS3 protein induced DNA damage in hepatocytes through double‐strand breaks 25. On the other hand, we previously reported that HCV core protein might disturb the DNA repair system in PH5CH8 cells by promoting microsatellite instability 26. In addition, we demonstrated that HCV NS5B protein increased the susceptibility of PH5CH8 cells to DNA double‐strand breaks 27. Further analysis is needed to identify which HCV protein is responsible for the surface expression of ULBP1 through the DNA damage response.

On the other hand, viruses are capable of evading the innate immune response by NK cells to establish persistent infection. HCMV was shown to inhibit the surface expression of ULBP1 and ULBP2 through binding with viral glycoprotein UL16 12. The HIV‐1 accessory protein Nef inhibited the surface expression of ULBP1 and ULBP2 in primary CD4^+^ T‐cells 28. We have not identified which HCV protein is responsible for the down‐modulation of NK cell functions. Moreover, in addition to the down‐modulation of NK cell functions by viral proteins, viruses may possess other ways of evading the innate immune response by NK cells 29. One strategy might be down‐modulating the NKG2D expression and NK cell functions by the soluble forms of NKG2D ligands. A previous study suggested that the soluble forms of ULBP2, MICA and MICB (sULBP2, sMICA and sMICB, respectively) were released during HIV‐1 infection 29. All three of these proteins—sULBP2, sMICA and sMICB—impaired NKG2D expression and the cytotoxicity of NK cells 29. In HCV‐induced liver diseases, the level of sMICA was elevated at the early stage of liver disease and was not correlated with the disease progression 30. In advanced human hepatocellular carcinoma, sMICA was responsible for the down‐modulation of NKG2D expression and NK cell functions 31. Another study suggested an additional possible strategy—namely, the down‐modulation of NK cell functions by the exosome released from the target cells 32. In that study, thermal and oxidative stresses were suggested to trigger the release of NKG2D ligand‐bearing immunosuppressive exosomes, thereby impairing NK cell functions. In the present study, we were not able to examine the roles of soluble NKG2D ligands and exosomes released from HCV‐infected cells against NK cells, since the culture medium was replaced with fresh medium before the co‐culture. Further analysis will be needed to examine the mechanism by which HCV evades the innate immune response by NK cells to establish persistent infection.

In the present study, we demonstrated that HCV induced the surface expression of ULBP1 in human hepatocytes (Fig. 2B–D). In addition, NK cells attacked HCV‐infected hepatocytes through the recognition of ULBP1 (Fig. 4F,G). From our results, we conclude that ULBP1 is the target of the NK cell‐mediated innate immune response in HCV‐infected human hepatocytes. Our results also suggest that ULBP1 is a potential target in immunotherapy against HCV‐induced liver diseases.

## Author contributions

HD and NK designed the research. HD performed most of the experiments. HI contributed pCX4bleo ULBP1. HD, HI, YU, SS and NK analyzed the data. HD and NK wrote the paper. All authors reviewed the manuscript.
